# Calcium-Rich Biochar Stimulates Salt Resistance in Pearl Millet (*Pennisetum glaucum* L.) Plants by Improving Soil Quality and Enhancing the Antioxidant Defense

**DOI:** 10.3390/plants11101301

**Published:** 2022-05-13

**Authors:** Kamal A. M. Abo-Elyousr, Magdi A. A. Mousa, Omer H. M. Ibrahim, Nouf Owdah Alshareef, Mamdouh A. Eissa

**Affiliations:** 1 Department of Arid Land Agriculture, Faculty of Meteorology, Environment and Arid Land Agriculture, King Abdulaziz University, Jeddah 80208, Saudi Arabia; ka@kau.edu.sa (K.A.M.A.-E.); mamousa@kau.edu.sa (M.A.A.M.); oabrahem@kau.edu.sa (O.H.M.I.); 2 Department of Biochemistry, Faculty of Science, King Abdulaziz University, Jeddah 80208, Saudi Arabia; noalshareef@kau.edu.sa; 3 Department of Soils and Water, Faculty of Agriculture, Assiut University, Assiut 71526, Egypt

**Keywords:** sandy soils, *Pennistum glaucum*, nutritive value, leaf biochemical, antioxidant enzymes

## Abstract

Shrimp waste is rich in organic compounds and essential plant nutrients, e.g., calcium (Ca), and converting these wastes to organic fertilizer is important for environmental preservation and to achieve sustainable agricultural management. In the current study, Ca-rich biochar was prepared from shrimp wastes (SWB) by pyrolysis at 300 °C. We hypothesized that the Ca-rich biochar will help in solving the problem of plant growth in saline soil by reducing sodium (Na) uptake and mitigating oxidative stress. The current study aimed to investigate the effect of SWB on the quality of saline sandy soil and the mechanism of salt resistance in pearl millet (*Pennisetum glaucum* L.). Pearl millet plants were planted in saline sandy soil (10 dS m^−1^) in wooden boxes (1.3 × 0.8 m size and 0.4 m height), and 5 doses (0, 1.0, 1.5, 2.0, and 2.5% (*w*/*w*)) of SWB were added. SWB application increased the soil quality and nutrient uptake by pearl millet plants. The highest rate of SWB increased the soil microbial biomass carbon and the activity of dehydrogenase enzyme by 43 and 47% compared to the control soil. SWB application reduced the uptake of sodium (Na^+^) and chloride (Cl^−^) and increased the K/Na ratio in the leaf tissues. SWB addition significantly increased the activity of antioxidant enzymes, e.g., ascorbate peroxidase (APX), polyphenol oxidase (PPO), and pyrogallol peroxidases (PPX). The application of 2.5% SWB to the saline soil increased the soluble carbohydrates and proline in plant leaves by 75 and 60%, respectively, and reduced the malondialdehyde (MDA) by 32% compared to the control. SWB enhanced the antioxidant defense and mitigated oxidative stress by improving the synthesis of osmoprotectants, e.g., soluble carbohydrates and proline. Sandy saline soils in arid and semiarid areas suffer greatly from low organic matter contents, which reduces the soil quality and increases the risk of salt during plant growth. The high organic matter and calcium content (30%) in the shrimp waste-derived biochar improved the quality of the saline sandy soil, reduced the uptake of toxic salts, and increased the quality of the forage material. The addition of recycled shrimp waste to saline low-fertility soils improves soil productivity and is safe for soil health.

## 1. Introduction

Pearl millet (*Pennisetum glaucum* L.) is an ideal model for the tolerance of several biotic and abiotic stresses and is considered a moderately salt-tolerant plant [1]. Furthermore, pearl millet has significant potential as a sustainable forage crop, especially in parched and semiparched areas that are threatened by environmental changes [2]. The dry stover and green fodder of pearl millet are used as animal feed [3]. The green forage of pearl millet is rich in protein and contains high concentrations of nutrients, e.g., calcium and phosphorus, with low concentrations of oxalic acid, which make it safer than other forage crops [3]. As a C_4_ plant, pearl millet is a promising feed crop that is hearty in many harsh environmental conditions and is characterized by its high photosynthetic efficiency and growth speed [4]. Pearl millet can grow in marginal lands and can provide a significant yield compared to other forage crops [5].

The challenges associated with soil salinity affect human food security in more than 100 countries, and approximately 800 million hectares of agricultural lands contain saline soils [6]. Salt toxicity inhibits plant growth and minimizes crop productivity and is considered one of the more difficult environmental challenges to address [7,8]. Salinity has harmful effects on plant growth by increasing the osmotic pressure in soil solution and nutrient imbalance; therefore, cell damage occurs as a result of oxidative stress [9,10]. The presence of salts on the surface of plant roots causes an additional imbalance in nutrient uptake [9,10,11]. The increase in sodium ions in saline soil solution disrupts potassium absorption [9,10]. Potassium is an essential plant nutrient that is involved in many biological reactions and is responsible for the regulation of more than 50 enzymes [8,11].

The application of organic fertilizers and amendments, e.g., manure, biochar, compost, and crop residues, could be an ideal solution to reclaim saline soils and develop a sustainable agroecosystem [12]. The incorporation of organic amendments in soil is a vital process and can lead to the conservation of soil organic matter and increase carbon sequestration in soil, which may help to reduce global warming [13]. In the last few decades, the application of organic amendments to saline soils has become a common practice to reduce the hazardous effect of salinity and improve plant growth [8,12]. The use of organic wastes as soil organic amendments is a crucial strategy for soil conservation and maintains sustainable agricultural development by recycling several kinds of wastes [8,14]. The application of organic materials to saline soils improves the physical and chemical properties of the soil and increases the activity of soil microbes and enzymes, thus improving plant growth and reducing the harmful effects of salts without any risks to the environment [15,16]. Organic wastes differ in their chemical composition according to their sources, and therefore, it is necessary to evaluate and measure the different effects of these organic wastes on the soil and plants before using them to avoid unexpected and negative effects and further land degradation [14,17].

Shrimp processing and consumption generate vast amounts of waste that is rich in organic matter and nutrients [18]. This shrimp waste is also considered a pollutant to the environment [18,19]. Shrimp wastes contain 34% crude protein, 29% ash, 14–30% calcium, and 2% phosphorus [18,20]. The conversion of shrimp wastes to organic fertilizers, such as biochar, is one of the ideal solutions for reducing pollution and organic wastes [18].

Although millet plants are resistant to high soil salinity (up to 15 dS m^−1^), the quality of the forage crop is severely reduced due to the harmful effect of salinity on the chemical composition of the plant [1,8]. Therefore, to address this issue, we attempted to develop soil amendments using decomposed shrimp wastes at high temperatures to produce biochar. The current study assumed that the application of shrimp waste-derived biochar, which is rich in Ca and organic matter, will reduce the negative effects of soil salinity on the growth, quality, and yield of pearl millet. We hypothesized that the Ca-rich biochar will reduce the Na uptake and minimize the oxidative stress caused by salt stress. Improving the soil health of low-fertility saline soils will improve plant resistance to salinity. Therefore, this study aimed to investigate the effect of shrimp waste-derived biochar on the health of saline sandy soil.

## 2. Materials and Methods

### 2.1. Preparation and Characterization of Shrimp Waste-Derived Biochar (SWB)

Shrimp wastes were collected from fish restaurants in Assiut, Egypt, and then air-dried and crushed. The dried wastes were pyrolyzed in a muffle furnace at 300 °C for 4 h and then ground to pass through a 2-mm sieve. Table 1 shows the main chemical characterizations of shrimp waste-derived biochar. The organic carbon and total nitrogen in SWB were measured by a CHNS analyzer (Elementar, Vario EL, Langenselbold Germany). SWB pH was determined in a 1:5 ratio (biochar: water) using a pH meter while salinity was determined by an electrical conductivity meter in a 1:5 ratio (biochar: water) based on the method outlined in Burt [21]. A sample of biochar (2 g) was digested by a mixture of H_2_SO_4_ and H_2_O_2_ based on the method of Parkinson and Allen [22], and then phosphorus, potassium, and calcium were determined in the digested sample. Phosphorus was determined using the ammonium molybdate reaction method; it was then measured using a spectrophotometer (Unico 2000 UV, Markham, Ontario, Canada) at 660 nm [21]. Potassium and calcium were measured by inductively coupled plasma spectroscopy (ICP−OES thermo iCAP 6000 series, Thermo Scientific, Kleve, Germany).

### 2.2. Experimental Setup and Treatments

A soil sample (0–20 cm) was collected from a saline sandy soil located at Assiut, Egypt, which is classified as Aridisols: Typic Torri psamments according to the Soil Survey Staff [23]. Table 2 shows some chemical and physical properties of the studied soil. Basic soil characterizations were conducted according to the methods of Burt [21]. The collected soil sample was air-dried and sieved through a 2 mm sieve. The current experiment was conducted in wooden boxes (1.3 × 0.8 m size and 0.4 m height) filled with the studied soil. Biochar was added to the soil at doses of 0, 10, 15, 20, and 25 g kg^−1^ (*w*/*w*) during the preparation of the soil. The treatments were arranged in a complete block design repeated five times. Pearl millet seeds (*Pennisetum glaucum* L. c.v. Shandweel 1) were planted in wooden boxes at a 15 × 20 cm distance with a plant density equal to 333,333 plants per hectare. Pearl millet plants were fertilized with 180:70:70 kg ha^−1^ of N:P:K. The plants were irrigated to near field capacity by ground water (3.2 dS m^−1^). After two months, the plants were harvested, and the growth parameters (plant height, leaf area index, fresh weight of shoot) were recorded. Based on the phonological scale, this stage was the maximum vegetative growth [8].

### 2.3. Soil and Plant Samples Analysis

Soil samples were collected at plant harvest to follow the changes in nutrient availability after the application of biochar. Organic carbon in the soil was measured by the dichromate oxidation method based on the Walkley–Black method [21]. The pH of the soil was determined in a 1:2 soil:water suspension by a pH meter while the soil salinity was measured in a soil paste extract by an electrical conductivity meter [21]. The available nitrogen was extracted from the soil by 2 M potassium chloride and then measured by the Kjeldahl method as in [21]. Available phosphorus was extracted from the soil samples by sodium bicarbonate solution (0.5 M, pH 8.5) according to the method used in Olsen et al. [21]. The extracted available phosphorus was determined by the ammonium molybdate reaction method and then measured by a spectrophotometer at 660 nm [21]. The available potassium was extracted by ammonium acetate and then measured by a flame photometer as previously described [21]. Soil microbial biomass carbon was extracted by the fumigation-extraction method [24], and then the total organic carbon was measured by a CHNS analyzer (Elementar, Vario EL, Langenselbold, Germany). The dehydrogenase activity in soil was measured by spectrophotometry at 485 nm after incubation of the soil sample with triphenyltetrazolium chloride for 24 h at 37 °C [25].

The plant samples of pearl millet were washed with distilled water and oven-dried at 70 °C, and then the dry weights were recorded. The oven-dried samples were ground and digested with a mixture of H_2_SO_4_ and H_2_O_2_, as described by Parkinson and Allen [22], to determine the concentrations of N, P, K, Na, Cl, and Ca. N, P, K, and Ca in the digested plant samples were determined according to the methods described in the biochar and soil analysis section. Na in the digested samples was determined by flame photometry while Cl was determined by titration with a standard solution of silver nitrate (0.01 M) according Burt [21]. Chlorophyll was extracted from fresh leaves by ethyl alcohol (95%) and then measured by spectrophotometry according to the method of Lichtenthaler [26]. The activity of antioxidant enzymes (pyrogallol peroxidase, ascorbate peroxidase, and polyphenol oxidase) was determined by the procedure described in Chen and Asada [27] and Sheyhakinia et al. [28]. Free proline was extracted from fresh pearl millet leaves (0.1 g) by 3% sulfosalicylic acid [29]. Soluble carbohydrates were extracted with ethanol (80%) and then measured by the anthrone reagent method [30]. Malondialdehyde (MDA) was measured based on the method of Madhava and Sresty [31].

### 2.4. Statistical Analysis

The data were checked for normality with the Shapiro–Wilk test. One-way ANOVA and Tukey’s test (*p* ≤ 0.05) were run to test the significance of differences between treatments. The SPSS 17.0 software package (SPSS, Chicago, IL, USA) was used in the statistical analysis of the data. All the data in tables and figures are means ± SE, *n* = 5.

## 3. Results

### 3.1. Soil Quality and Nutrients Uptake

Shrimp waste-derived biochar (SWB) significantly (*p* ≤ 0.05) improved the soil quality and increased nutrient availability (Table 3). All the studied rates of biochar increased the soil organic carbon (SOC); on the other hand, SWB addition significantly (*p* ≤ 0.05) reduced the soil pH. The highest dose of SWB (2.5%) increased the soil organic carbon (SOC) by 10% and reduced the pH by 6.5% compared to the control.

The SOC of the studied soil ranged between 6.2–6.8 g kg^−1^, and the maximum value was found in the soil treated with the highest dose of SWB (2.5%) while the lowest value was found in the control soil. The pH of the studied soil ranged from 8.02–7.48, and the maximum value was found in the control while the lowest pH value was found in the soil amended with 2.0% SWB. All the studied rates of SWB increased the soil’s available N, P, and K levels. The application of 2.5% SWB increased the soil’s available N, P, and K by 25.0, 32.1, and 7.2%, respectively, compared to the control soil. The application of 2.5% SWB increased the soil microbial biomass carbon and the activity of dehydrogenase enzyme by 43 and 47% compared to the control soil.

Table 4 shows the effect of SWB on the nutrient concentrations in the chutes of the pearl millet. The application of SWB had significant effects on the nutrient concentrations in the chutes of the pearl millet. SWB increased the N, P, K, and Ca concentrations and significantly reduced the Na and Cl concentrations in the chutes of the pearl millet. The application of the highest dose of SWB (2.5%) increased the N, P, K, and Ca concentrations by 40.0, 40.0, 60.0, and 55.6% and reduced the Na and Cl by 27.5 and 66.7% compared to the control. Furthermore, the application of SWB significantly increased the ratio of K/Na. The highest value of K/Na was found in the soil treated with the highest dose of SWB (2.5%) while the lowest value was found in the plants grown in the saline soil without the addition of SWB.

### 3.2. Growth and Forage Quality of Pearl Millet

The response of the pearl millet’s growth to the applied shrimp waste-derived biochar is presented in Table 5. SWB application significantly improved the growth of pearl millet. All the studied rates of SWB increased the chlorophyll (a and b), leaf area, plant height, and fresh and dry weight of chutes. The application of the highest dose of SWB (2.5%) increased the chlorophyll (a and b), leaf area, plant height, and fresh and dry chutes by 22.2, 32.3, 32.7, 25.0, 30.0, and 55.0%, respectively, compared to the control.

The application of SWB significantly improved the quality of forage (Table 6). All the studied rates of SWB increased the leaf/stem, crude protein, and ash contents, and significantly reduced the crude fiber content. The highest dose of SWB (2.5%) improved the leaves/stems, crude protein, and ash by 14.5, 29.4, and 51.2% and reduced the crude fiber by 16.7% compared to the control.

### 3.3. Activities of Antioxidant Enzymes

**Figure 1:**
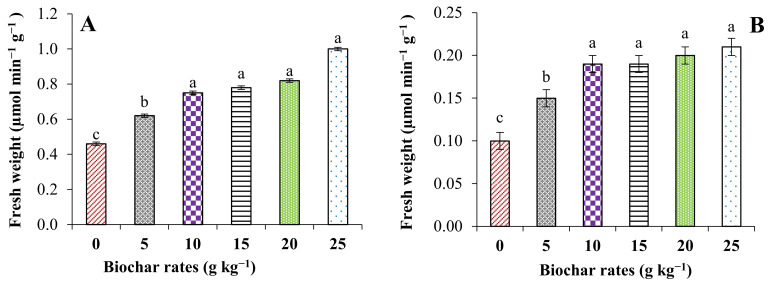

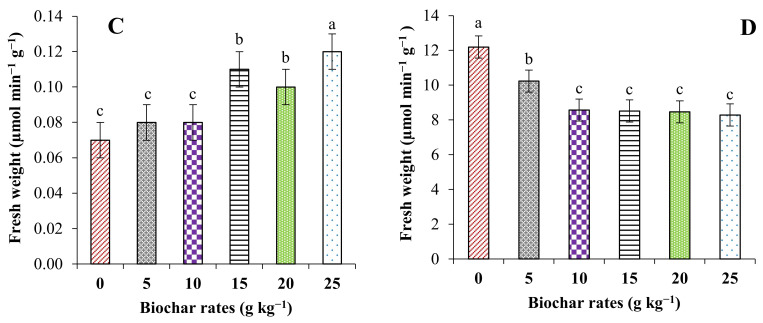
Effect of SWB on the activities of some antioxidant enzymes. (**A**): ascorbate peroxidase, (**B**): polyphenol oxidase, (**C**): pyrogallol peroxidases), and (**D**): malondialdehyde. The results are based on the fresh weight. Means (±SE) followed by different letters are significantly different according to Tukey’s test (*p* ≤ 0.05).

The activity of antioxidant enzymes in the leaves significantly responded to the levels of shrimp waste-derived biochar (SWB) added to the saline soil. Significant increases were observed in ascorbate peroxidase (APX), polyphenol oxidase (PPO), and pyrogallol peroxidases (PPX) in the leaf tissues of pearl millet when SWB was applied to saline sandy soil compared to the control (Figure 1). The application of the highest dose of SWB (2.5%) increased AXP, PPO, and PPX by 117.4, 110, and 71.4%, respectively, compared to the control.

**Figure 2:**
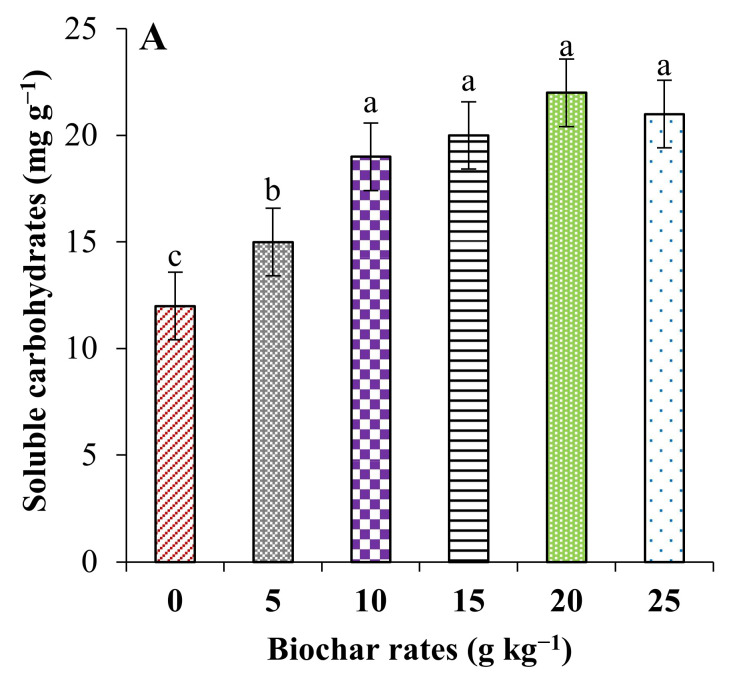

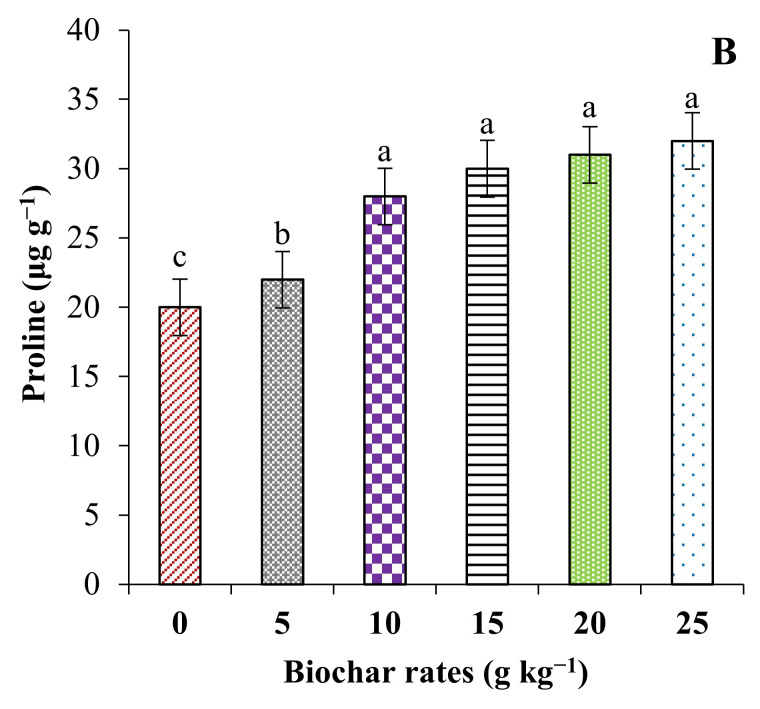
Effect of SWB on soluble carbohydrates and proline in the leaves of pearl millet plants. (**A**): soluble carbohydrates and (**B**): proline. Means (±SE) followed by different letters are significantly different according to Tukey’s test (*p* ≤ 0.05).

SWB application caused significant increases in osmoprotectants, e.g., soluble carbohydrates and proline, in the leaves of pearl millet plants; on the other hand, SWB significantly reduced the concentrations of malondialdehyde (MDA) in the leaves of plants (Figure 2). Plants grown in saline soils without the addition of SWB contained the highest concentration of MDA and the lowest concentrations of osmoprotectants. The application of the highest dose of SWB (2.5%) increased soluble carbohydrates and proline by 75.0 and 60.0%, respectively, and reduced MDA by 32.0% compared to the control.

## 4. Discussion

The lowest growth of pearl millet plants was found in the saline soil without biochar addition. The growth and forage quality of pearl millet plants were severely reduced due to the harmful effect of salinity on the chemical composition of the plant [1,8]. Sodium (Na^+^) ions predominate on the surfaces of mineral and organic compounds in saline soils, causing increases in the concentration of Na^+^ in the soil solution, thus having a direct effect on plant root hairs [32]. The presence of salts on the surface of the plant root hairs leads to an imbalance in the plant’s ability to absorb nutrients; for example, the presence of high concentrations of Na^+^ disrupts K^+^ uptake by the plant [8,10,32]. A potassium deficiency in the plant leads to an imbalance in the regulation of more than 50 enzymes, and thus, many vital processes in the plant are affected [11]. Sodium toxicity in the rhizosphere restricts K uptake and negatively affects the integrity of the plant membranes and causes damage, which ultimately alters their selectivity [32]. The negative effects of imbalances of nutrients and toxicity of Na^+^ on reducing plant growth have been reported in some previous studies, e.g., Ali et al. [8] and Ding et al. [33].

The limited growth of pearl millet in saline sandy soil results from several hindrances, including the salinity and low fertility of sandy soils. The application of shrimp waste-derived biochar mitigated the adverse effect of soil salinity and improves the yield and forage quality of pearl millet. Biochar application at a dose of 2.5% (*w/w*) increased the leaf/stem, crude protein, and ash contents by 14.5, 23.5, and 51.2%, respectively, and reduced the crude fiber content by 16.7% in comparison with the nonamended soil. Furthermore, the application of the highest dose of biochar increased the plant height, leaf area index, and fresh and dry shoot by 25.0, 32.7, 30.0, and 55.0%, respectively, compared to the control. The obtained results confirm the positive role of biochar in improving the growth and quality of forage for pearl millet crops. We assume that the addition of biochar reduced the negative effect of Na^+^ and restored the balance in nutrients, which encouraged the growth of plants. Shrimp waste-derived biochar is rich in essential nutrients, e.g., N, P, K, and Ca (Table 1). The high concentrations of Ca (30%) in shrimp waste-derived biochar have a positive role in reducing Na^+^ toxicity in plant growth [34]. Calcium reduces the permeability of the cell membrane against Na^+^ and prevents its accumulation in the plant cell and thus restores the ionic balance within the plant cells [20,34,35]. Therefore, the presence of high concentrations of calcium in the soil soliton protects the pearl millet against soil salinity. The addition of biochar increased the plant absorption of calcium and potassium and decreased the absorption of sodium and chloride, which supports this assumption outlined in this study. Among other supporting evidence for this hypothesis, the increased K/Na ratio in the leaf tissues is a result of biochar addition. The positive role of shrimp waste-derived biochar in reducing the toxicity of salts was confirmed by Kazemi et al. [18].

It is well established that salt stress reduces the availability and uptake of nutrients, which negatively affects plant growth [8,36]. Conversely, the addition of shrimp waste-derived biochar increased the availability and uptake of nutrients, e.g., N, P, K, and Ca. The application of rich carbon materials, such as biochar, to sandy soil improves the soil content in organic materials and improves the soil quality, which results in an increase in the availability and uptake of nutrients [12,37]. The cycle of nutrients and their availability to the plant is related to the activity of soil microorganisms and the activity of soil enzymes [16,38]. Sandy soils have low contents of organic matter, and therefore, the activity of soil enzymes and microorganisms decreases, and the seriousness of the problem increases in saline soils [12,16,39]. The findings of the current study showed that the application of biochar significantly improved the activity of dehydrogenase enzymes and enhanced the soil microbial biomass. Dehydrogenase activity, as an intracellular enzyme, is a good indicator in the evaluation of soil quality because dehydrogenase is directly connected to the activity of soil microorganisms and the stability of soil organic matter [40,41]. Dehydrogenase activity in sandy soil increased by more than 50% when the soil was amended with wheat straw-derived biochar [42]. We assume that biochar addition led to an improvement in the physicochemical characteristics of the saline sandy soil and thus increased the activity of soil enzymes and the activity of soil microbes, which led to an increase in the availability of nutrients and their absorption by pearl millet plants. Biochar contains stable organic compounds and thus improves the soil physicochemical properties, as reported by many previous studies, e.g., Liu et al. [16] and Yao et al. [38].

Soil salinity has detrimental effects on plant growth by increasing oxidative stress in plant cells [10,43,44,45,46,47]. The activity of antioxidant enzymes in pearl millet plants was increased by the application of shrimp waste-derived biochar. The applica-tion of biochar regulates the synthesis of antioxidant enzymes in pearl millet plants and therefore improves salt stress resistance [43,44]. The positive role of biochar in re-ducing oxidative stress was attributed to the reduction in malondialdehyde (MDA) concentrations, resulting in higher growth [43,45,48,49,50,51]. The application of the highest dose of shrimp waste-derived biochar (2.5%) increased the soluble carbohydrates and proline by 75 and 60% and reduced MDA by 32% in comparison with the nonamended soil. The application of shrimp waste-derived biochar alleviates oxidative stress and osmoprotectants, e.g., soluble carbohydrates and proline. Increases in the activity of antioxidant enzymes and the synthesis of osmoprotectants as a result of biochar addi-tion have been reported by Alam et al. [46] and Liu et al. [52].

## 5. Conclusions

Recycling shrimp wastes to biochar preserves the environment and achieves sustainable agricultural development. In the current study, shrimp wastes were converted to biochar by pyrolysis at 300 °C. The produced biochar contains high organic carbon and nutrients, especially calcium (30%, *w*/*w*). Shrimp waste-derived biochar was added to saline sandy soil to increase the productivity and quality of pearl millet plants. The application of shrimp waste-derived biochar increases the growth, yield, and forage quality of pearl millet. Shrimp waste-derived biochar enhances salt tolerance and reduces the negative effects of soil salinity by improving the antioxidant strength and increasing the synthesis of osmoprotectants. Furthermore, shrimp waste-derived biochar mitigates oxidative stress by reducing the accumulation of sodium and chloride in plant leaves. The low organic matter content in sandy saline soils in arid and semiarid areas increases the toxicity of sodium and other salts. Shrimp waste-derived biochar increases the soil quality by improving the soil organic matter and activity of soil enzymes and soil microbial biomass. The addition of wastes to agricultural lands needs further study to determine its effects on soil health before use as soil amendments. The findings of the current study confirm the positive role of shrimp waste-derived biochar in improving the quality of saline soils. More studies are required to explore the effect of manufacturing this type of biochar at different temperatures. Moreover, several studies must be conducted to explore the behavior of shrimp wastes in different types of soils and when it is mixed with other soil amendments.

## Figures and Tables

**Table 1:** Chemical characterization of shrimp waste-derived biochar.

N (%)	P (%)	K (%)	Ca (%)	Organic-C (%)	pH_1:5_	EC_1:5_ (dS m^−1^)	Yield (%)
6.4 ± 0.1	5.2 ± 0.2	3.2 ± 0.1	30 ± 2	40 ± 3	6.20 ± 0.07	3.50 ± 0.05	87

**Table 2:** Basic soil properties.

Soil Properties	Value
Clay (g kg^−1^)	20 ± 2
Silt (g kg^−1^)	80 ± 5
Sand (g kg^−1^)	900 ± 23
Texture	Sandy
CaCO_3_ (g kg^−1^)	70 ± 6
CEC (cmol kg^−1^)	15 ± 0
pH (1:2)	8.00± 0.05
Salinity (dS m^−1^)	10.0 ± 0.4
Organic carbon (g kg^−1^)	11.8 ± 0.1
Total N (mg kg^−1^)	350 ± 8
Total P (mg kg^−1^)	1780 ± 35
Total K (mg kg^−1^)	880 ± 20
Available N (mg kg^−1^)	40 ± 4
Available P (mg kg^−1^)	5.8 ± 0.2
Available K (mg kg^−1^)	200 ± 12

**Table 3:** Effect of SWB on soil quality.

SWB Rates (g kg^−1^)	pH	Organic-C (g kg^−1^)	Available-N (mg kg^−1^)	Available-P (mg kg^−1^)	Available-K (mg kg^−1^)	MBC (mg C kg^−1^)	DHS (μg TPF g^−1^ h)
0	8.02 ± 0.1 ^a^	6.2 ± 0.2 ^b^	48 ± 0.1 ^b^	5.6 ± 0.1 ^b^	250 ± 0.07 ^b^	300 ± 8 ^d^	150 ± 16 ^d^
5	8.00 ± 0.1 ^a^	6.3 ± 0.2 ^b^	55 ± 0.2 ^a^	6.8 ± 0.2 ^a^	260 ± 0.03 ^a^	350 ± 17 ^c^	170 ± 16 ^c^
10	7.73 ± 0.2 ^b^	6.5 ± 0.2 ^ab^	56 ± 0.1 ^a^	7.0 ± 0.3 ^a^	265 ± 0.01 ^a^	380 ± 8 ^b^	200 ± 16 ^b^
15	7.64 ± 0.1 ^b^	6.6 ± 0.1 ^a^	55 ± 0.2 ^a^	7.1 ± 0.3 ^a^	268 ± 0.04 ^a^	390 ± 15 ^b^	205 ± 16 ^b^
20	7.48 ± 0.2 ^c^	6.7 ± 0.2 ^a^	57 ± 0.1 ^a^	7.5 ± 0.2 ^a^	270 ± 0.01 ^a^	400 ± 12 ^b^	215 ± 16 ^a^
25	7.52 ± 0.2 ^c^	6.8 ± 0.1 ^a^	60 ± 0.1 ^a^	7.4 ± 0.3 ^a^	268 ± 0.05 ^a^	430 ± 16 ^a^	220 ± 16 ^a^

MCB = microbial biomass, DHS = dehydrogenase enzyme. Means (±SE) followed by different letters are significantly different according to Tukey’s test (*p* ≤ 0.05).

**Table 4:** Effect of SWB on the nutrient concentrations in plant shoots.

Biochar Rates (g kg^−1^)	N (%)	P (%)	K (%)	Ca (%)	Na (%)	Cl (%)	K/Na
0	1.5 ± 0.1 ^b^	0.18 ± 0.04 ^b^	1.7 ± 0.1 ^b^	1.0 ± 0.1 ^b^	1.38 ± 0.07 ^a^	0.21 ± 0.07 ^a^	1.23 ± 0.07 ^b^
5	3.1 ± 0.1 ^a^	0.31 ± 0.05 ^a^	2.7 ± 0.2 ^a^	2.3 ± 0.2 ^a^	1.25 ± 0.03 ^b^	0.15 ± 0.03 ^b^	2.16 ± 0.07 ^a^
10	3.3 ± 0.2 ^a^	0.35 ± 0.06 ^a^	2.8 ± 0.1 ^a^	2.5 ± 0.3 ^a^	1.22 ± 0.01 ^c^	0.12 ± 0.01 ^c^	2.30 ± 0.07 ^a^
15	3.4 ± 0.1 ^a^	0.34 ± 0.07 ^a^	2.8 ± 0.2 ^a^	2.7 ± 0.3 ^a^	1.11 ± 0.04 ^c^	0.13 ± 0.04 ^c^	2.52 ± 0.07 ^a^
20	3.7 ± 0.2 ^a^	0.38 ± 0.09 ^a^	3.0 ± 0.1 ^a^	2.8 ± 0.2 ^a^	1.10 ± 0.01 ^c^	0.09 ± 0.01 ^c^	2.73 ± 0.07 ^a^
25	3.5 ± 0.2 ^a^	0.39 ± 0.05 ^a^	3.3 ± 0.1 ^a^	2.8 ± 0.3 ^a^	1.00 ± 0.05 ^c^	0.07 ± 0.05 ^c^	3.30 ± 0.07 ^a^

Means (±SE) followed by different letters are significantly different according Tukey’s test (*p* ≤ 0.05).

**Table 5:** Effect of SWB on the growth of pearl millet.

Biochar Rates (g kg^−1^)	Chlorophyll-a (mg g plant^−1^)	Chlorophyll-b (mg g plant^−1^)	Plant Height (cm)	Leaf Area Index (m^2^ m^–2^)	Shoot Fresh Weight	Shoot Dry Weight
0	2.70 ± 0.09 ^b^	1.55 ± 0.10 ^c^	68 ± 2 ^c^	5.5 ± 0.3 ^c^	200 ± 12 ^d^	100 ± 6 ^d^
5	3.10 ± 0.10 ^a^	1.85 ± 0.05 ^b^	75 ± 2 ^b^	6.7 ± 0.5 ^b^	220 ± 8 ^c^	123 ± 5 ^c^
10	3.20 ± 0.10 ^a^	1.90 ± 0.12 ^b^	80 ± 4 ^a^	7.0 ± 0.6 ^ab^	235 ± 15 ^b^	130 ± 6 ^b^
15	3.15 ± 0.14 ^a^	2.00 ± 0.14 ^a^	82 ± 3 ^a^	7.2 ± 0.5 ^a^	250 ± 22 ^a^	150 ± 8 ^a^
20	3.20 ± 0.09 ^a^	2.10 ± 0.17 ^a^	83 ± 2 ^a^	7.1 ± 0.4 ^ab^	255 ± 20 ^a^	154 ± 4 ^a^
25	3.30 ± 0.11 ^a^	2.05 ± 0.11 ^a^	85 ± 2 ^a^	7.3 ± 0.4 ^a^	260 ± 18 ^a^	152 ± 5 ^a^

Means (±SE) followed by different letters are significantly different according Tukey’s test (*p* ≤ 0.05).

**Table 6:** Effect of SWB on the forage quality of pearl millet.

Biochar Rates (g kg^−1^)	Leaves/Stems	Crude Protein (%)	Ash (%)	Crude Fiber (%)
0	0.49 ± 0.2 ^b^	17 ± 1 ^c^	8.0 ± 0.2 ^d^	35 ± 2 ^a^
5	0.55 ± 0.3 ^a^	19 ± 1 ^b^	10.0 ± 0.4 ^c^	32 ± 2 ^b^
10	0.54 ± 0.1 ^a^	21 ± 2 ^ab^	11.0 ± 0.3 ^b^	31 ± 2 ^b^
15	0.56 ± 0.2 ^a^	21 ± 2 ^ab^	11.6 ± 0.1 ^ab^	32 ± 2 ^b^
20	0.57 ± 0.2 ^a^	23 ± 2 ^a^	12.0 ± 0.2 ^a^	30 ± 2 ^b^
25	0.55 ± 0.2 ^a^	22 ± 2 ^a^	12.1 ± 0.2 ^a^	30 ± 2 ^b^

Means (±SE) followed by different letters are significantly different according Tukey’s test (*p* ≤ 0.05).

## Data Availability

Data is contained within the article.
